# Neonatal Diabetes Mellitus

**DOI:** 10.3389/fped.2020.540718

**Published:** 2020-09-30

**Authors:** Jacques Beltrand, Kanetee Busiah, Laurence Vaivre-Douret, Anne Laure Fauret, Marianne Berdugo, Hélène Cavé, Michel Polak

**Affiliations:** ^1^Paediatric Endocrinology, Gynaecology and Diabetology, Necker–Enfants Malades University Hospital, Assistance Publique-Hôpitaux de Paris, IMAGINE Institute, ENDO-European Reference Network Team, Paris, France; ^2^Faculty of Medicine, Université de Paris, Paris, France; ^3^INSERM U1016, Cochin Institute, Paris, France; ^4^Paediatric Endocrinology, Diabetology and Obesity Unit, Lausanne University Hospital, University of Lausanne, Lausanne, Switzerland; ^5^Inserm UMR-1018-CESP, Necker–Enfants Malades University Hospital Paedopsychiatry Department, Cochin University Hospital Paediatrics Department, Institut Universitaire de France, Assistance Publique-Hôpitaux de Paris, Université de Paris, Paris, France; ^6^Genetics Department, Robert-Debré University Hospital, Assistance Publique-Hôpitaux de Paris, Paris, France; ^7^INSERM U1138, Cordeliers Research Centre, Paris, France

**Keywords:** neonatal diabetes mellitus, chromosome 6q24 abnormality, associated malformations, neuropsychological disorder, KCNJ11 (Kir6.2), ABCC8, sulfonylurea receptor (SUR1)

## Abstract

Neonatal Diabetes (ND) mellitus is a rare genetic disease (1 in 90,000 live births). It is defined by the presence of severe hyperglycaemia associated with insufficient or no circulating insulin, occurring mainly before 6 months of age and rarely between 6 months and 1 year. Such hyperglycaemia requires either transient treatment with insulin in about half of cases, or permanent insulin treatment. The disease is explained by two major groups of mechanism: malformation of the pancreas with altered insulin-secreting cells development/survival or abnormal function of the existing pancreatic β cell. The most frequent genetic causes of neonatal diabetes mellitus with abnormal β cell function are abnormalities of the 6q24 locus and mutations of the *ABCC8* or *KCNJ11* genes coding for the potassium channel in the pancreatic β cell. Other genes are associated with pancreas malformation or insufficient β cells development or destruction of β cells. Clinically, compared to patients with an *ABCC8* or *KCNJ11* mutation, patients with a 6q24 abnormality have lower birth weight and height, are younger at diagnosis and remission, and have a higher malformation frequency. Patients with an *ABCC8* or *KCNJ11* mutation have neurological and neuropsychological disorders in all those tested carefully. Up to 86% of patients who go into remission have recurrent diabetes when they reach puberty, with no difference due to the genetic origin. All these results reinforce the importance of prolonged follow-up by a multidisciplinary pediatric team, and later doctors specializing in adult medicine. 90% of the patients with an *ABCC8* or *KCNJ11* mutation as well as those with 6q24 anomalies are amenable to a successful switch from insulin injection to oral sulfonylureas.

## Definition

Diabetes mellitus in very young children or neonatal diabetes is a rare genetic disease (minimal incidence: 1 in 90,000 live births) with variations within different ethnic groups (1–3). It is defined by the presence of severe hyperglycaemia requiring treatment and occurs between the neonatal period and infancy. It occurs mainly before 6 months of age (155/173 probands in our published cohort) and rarely between 6 months and 1 year (18/173) (4). In the Finnish population for example, after 6 months of age, patients with diabetes had high HLA risk genotypes and islet autoantibodies, reflecting the autoimmune character of diabetes (5). This hyperglycaemia is associated with insufficient or no circulating insulin (3). Two clinical forms have been distinguished, based on the duration of the treatment: a so called “transient form” and a permanent form.

**Figure 1:**
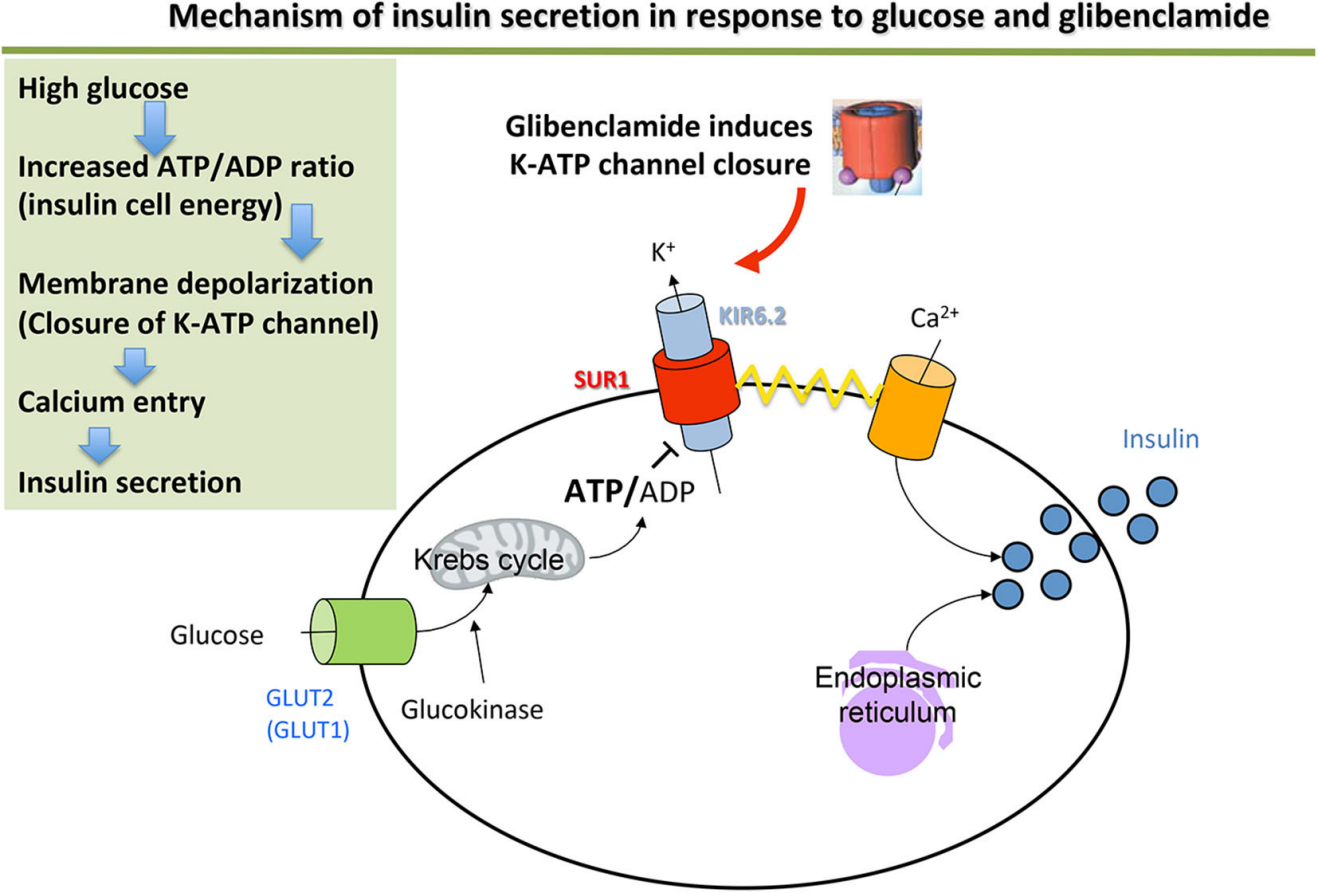
Mechanism of insulin secretion in response to glucose and glibenclamide.

The disease is explained by two major groups of mechanism: malformation of the pancreas or abnormal function of the pancreatic β cell that secretes insulin (by poor insulin cell mass development or malfunction of a cell component or by destruction of the β cell) (Table 1) (see Figure 1 for the normal functioning of the β cell).

**Table 1:** Genetic causes of monogenic neonatal diabetes based on physiopathological mechanisms [excluding 6q24 locus abnormalities (MIM *601410, *603044, and *612192)].

**Gene/Protein**	**Function**	**Locus**	**Transmission mode**	**Type of diabetes**	**Reference OMIM numbers**
**BETA CELL FUNCTION ABNORMALITY**					
ABCC8/SUR1	K_ATP_ channel/insulin secretion	11p15.1	Dominant	PND/TND/iDEND/DEND	MIM *600509
KCNJ11/Kir6.2	K_ATP_ channel/insulin secretion	11p15.1	Dominant	PND/TND/iDEND/DEND	MIM *600937
INS/Insulin	Hormone	11p15.5	Rare Recessive	Isolated TND/PND	MIM *176730
GCK/Glucokinase	Glucose metabolism	7p15.3-p15.1	Recessive/Dominant	Heterozygous: MODY2 Homozygous: PND	MIM *138079
SLC2A2/GLUT2	Membrane receptor	3q26.1-q26.2	Recessive	PND/TND + Fanconi-Bickel syndrome (glycogenosis) Proximal tubulopathy + small size + rickets + abnormality of glucose and galactose metabolism	MIM *138160
SLC19A2	Thiamine transporter	1q23.3	Recessive	Rogers Syndrome: Thiamine-sensitive megaloblastic anemia + diabetes + perceptive deafness ± PND	MIM *603941
**ENDOCRINE PANCREAS DEVELOPMENT ABNORMALITY**					
GATA6/GATA6	Transcription factor	18q11.1-q11.2	Dominant	PND by pancreas agenesis/hypoplasia + congenital cardiopathy + biliary tract abnormalities	MIM *601656
GLIS3/Zinc finger protein, GLIS3	Transcription factor	9p24.2	Recessive	PND + congenital hypothyroidism ± progressive hepatic fibrosis ± cystic renal dysplasia ± congenital glaucoma	MIM *610192
HNF1β/HNF1β	Transcription factor	17q12	Dominant	MODY5 or TND + pancreatic hypoplasia + renal cyst	MIM *189907
NEUROD1/BETA2	Transcription factor	2q31.3	Recessive/Dominant	Heterozygous: MODY6 Homozygous: PND + cerebellar hypoplasia + visual defect + perceptive deafness	MIM *601724
NEUROG3/Neurogenin3	Transcription factor	10q21.3	Recessive	Homozygous hypomorphic mutation: congenital malabsorption diarrhea + late-onset diabetes (8 years) Homozygous nonsense mutation: PND + congenital malabsorption diarrhea	MIM *604882
PAX6/aniridia type II protein, Pax6	Transcription factor	11p13	Recessive	PND + microphthalmia + cerebral malformation	MIM *607108
PDX1 (or IPF1)/Pancreas/duodenum homeobox protein 1	Transcription factor	13q12.1	Recessive/Dominant	Heterozygous: MODY4 Homozygous nonsense mutation: PND by agenesis/hypoplasia of the pancreas Homozygous hypomorphic mutation: PND by hypoplasia of the pancreas	MIM *600733
PTF1A/Pancreas Transcription Factor 1	Transcription factor	10p12.2	Recessive	PND by agenesis of the pancreas + cerebellar agenesis	MIM *607194
RFX6/Rfx6	Transcription factor	6q22.1	Recessive	Martinez-Frias Syndrome: Pancreatic hypoplasia + intestinal atresia with diarrhea + agenesis/hypoplasia of the gall bladder	MIM *612659
CNOT1	Transcriptional repressor	16q21	*De novo* specific mechanism of the mutation	Pancreatic agenesis + holoprosencephaly	MIM *604917
**DESTRUCTION/ENDOPLASMIC RETICULUM STRESS WITH LOW INSULIN CELL MASS OR DESTRUCTION OR EARLY IMMUNE DESTRUCTION**					
**OF THE BETA CELLS**					
INS/Insulin	Hormone	11p15.5	Dominant	PND	MIM *176730
EIF2AK3/EIF2AK3	Enzyme	2p11.2	Recessive	Wolcott Rallison Syndrome: PND + epiphyseal dysplasia	MIM *604032
IER3IP1/immediate early response 3-interacting protein 1	Endoplasmic reticulum protein	18q12	Recessive	PND + microcephaly + lissencephaly + epilepsy	MIM *300292
FOXP3/Forkhead box protein P3	Transcription factor (Forkhead domain)	Xp11.23	X-linked recessive	IPEX syndrome: Immunodysregulation Polyendocrinopathy Enteropathy X-linked: PND + increased IgE levels	MIM *300292
STAT3	Transcription factor	17q21.2	Dominant	Autoimmune disease, multisystem PND	MIM *102582
WFS1/Wolframin	Transmembrane protein of the endoplasmic reticulum	4p16.1	Recessive	Wolfram Syndrome: PND + optic atrophy ± diabetes insipidus ± deafness (DIDMOAD)	MIM *222300

*ND, neonatal diabetes; PND, permanent neonatal diabetes; TND, transient neonatal diabetes; DEND, Developmental delay, Epilepsy and Neonatal Diabetes; iDEND, intermediate DEND = DEND without epilepsy; MODY, Maturity Onset Diabetes of the Young*.

## Genetic Causes

### Abnormal β Cell Function

The most frequent genetic causes of neonatal diabetes with normal pancreas morphology are abnormalities of the 6q24 locus and mutations of the genes coding for the ATP-dependent potassium channel.

#### 6q24 (MIM#601410 and 603044)

The first genetic causes identified were abnormalities of the 6q24 locus, which include paternal uniparental disomy of 6q24 (pUPD6), partial duplication of paternal 6q24 and relaxation of the maternal 6q24 imprinted locus. This locus contains a CpG island, presenting differential methylation depending on the parental origin (non-methylation on the paternal allele, methylation on the maternal allele) (6). To date, the methylation abnormality has not been found in the parents of affected children. Methylation is used to down-regulate gene transcription of the methylated allele. All these abnormalities lead to over-expression of imprinted genes located in 6q24, such as PLAGL1/ZAC (pleiomorphic adenoma gene-like 1) and HYMAI (Hydatidiform mole-associated and imprinted transcript), which are the most “likely” candidate genes (6–8). PLAGL-1 codes for a transcription factor involved in regulation of stopping the cell cycle and apoptosis and in induction of the receptor 1 gene for human pituitary adenylate cyclase-activating polypeptide (PACAP1, which is a potent stimulant of insulin secretion). The function of the HYMAI gene is unknown (9). The mechanism responsible for the diabetes could be linked to a developmental defect in the β cells but the fact that remission of the diabetes occurs means that an abnormality in β cell function cannot be ruled out (10). The 6q24 abnormalities are associated with “transient” neonatal diabetes (7, 8, 11).

**The ZFP57 gene (MIM** ^*^612192) is involved in maintaining methylation of the DNA during the very early stages of embryogenesis. It is localized at 6p22.1. Homozygous mutations leading to a lack of protein or non-functional protein are associated with widespread DNA hypomethylation, including hypomethylation of the 6q24 locus (12). However, there are patients who have a 6q24 methylation abnormality not due to mutations of this gene (12).

#### Mutations of the ABCC8 and KCNJ11 Genes Coding for the K_ATP_ Channel: (MIM ^*^600509 and ^*^600937)

The ATP-dependent potassium channel (K_ATP_ channel) plays a central role in stimulating insulin secretion by the pancreatic β cell in response to glucose. At low blood sugar levels (e.g., fasting), the K_ATP_ channels are open (activated) and their activity maintains a hyperpolarized resting membrane potential (around −70 mV). A rise in blood sugar level (e.g., post-prandial) causes increased passage of glucose into the β cell. Glucose enters the glycolysis pathway, which increases the intracellular ATP concentration. This causes the K_ATP_ channels to close (inhibition), which leads to the intracellular potassium accumulation that causes membrane depolarization. This depolarization activates the voltage-dependent calcium channels, leading to Ca^2+^ ions entering the β cell, then enabling exocytosis of the secretion vesicles and release of insulin into the bloodstream (Figure 1).

The K_ATP_ channel is an octamer formed from two types of subunits: the Kir6.2 subunits form the channel selective for the incoming corrective potassium enclosed in SUR1 ion-channel regulator subunits (13, 14). They are coded by the *KCNJ11* and *ABCC8* genes, respectively.

Activating mutations in one of these two genes are responsible for neonatal diabetes with normal pancreas morphology (15–17). They result in the K_ATP_ channel remaining permanently open, so that it no longer controls membrane potential in response to glucose and therefore blocks the event cascade that leads to insulin release.

#### Mutations of the Insulin Gene (INS) (MIM ^*^176730)

The third cause of neonatal diabetes, by frequency, is mutations of the insulin gene (*INS*). The majority are heterozygous mutations affecting the structure of preproinsulin; these are transmitted in an autosomal dominant manner (18, 19). The abnormal proinsulin undergoes degradation in the endoplasmic reticulum, leading to severe endoplasmic reticulum (ER) stress and β cell death. This process has been described in mouse models (20) and in man (21, 22). Recent evidence suggests that *INS* mutations do not necessarily lead to beta-cell death but rather the chronic ER stress interferes with beta-cell growth and development (23).

Some mutations alter expression of the protein. They are transmitted in a recessive manner, in the majority of cases in consanguineous families. These mutations affect the insulin promoter directly of by mutation in factor that enhances its activity (24, 25).

#### Mutations of the Glucokinase Gene (MIM ^*^138079)

Glucokinase is responsible for the first step of glucose metabolism in the β cell. It acts as a “sensor” of blood glucose, making it possible to control the quantity of insulin secreted. Nonsense mutations of the glucokinase gene cause MODY 2 (Maturity onset diabetes in the youth type 2), which usually presents as moderate hyperglycaemia (26). Transmission is heterozygous. In the homozygous state, these nonsense mutations cause neonatal diabetes by complete deficiency of glucokinase-mediated glycolysis (27). This is not a frequent cause of neonatal diabetes (28, 29). However, an assay of the fasting blood glucose concentration is required from both parents, particularly if there is a history of gestational diabetes. The discovery of discreet glucose intolerance in both parents should therefore lead to a search for glucokinase gene mutations.

### Abnormal Pancreas Morphology

Several genes are linked to neonatal diabetes with abnormal pancreas morphology and precise description is beyond the scope of this chapter (see Table 1 for a brief information). These genes are involved in development of the pancreas at various stages in early morphogenesis. These neonatal diabetes cases may be associated with a deficiency of the exocrine pancreas, based on the severity of pancreatic damage or to other congenital malformations. Mutation of the RFX-6 gene deserves a specific comment. The RFX-6 transcription factor is involved in the differentiation of beta-cells in the pancreas during embryonic development of the pancreas. It is also expressed in mature cells where it has a role in regulating insulin transcription and secretion. It actually controls the expression and activation of calcium channels and its inactivation alters insulin secretion in response to glucose. A few cases of neonatal diabetes have been reported. Patients display developmental abnormalities of the pancreas and of the digestive tract. The mechanism is linked to both a developmental and a functional disorder of the endocrine pancreas. Transmission is autosomal recessive (Table 1).

### Autoimmune Neonatal Diabetes Mellitus

Most patients diagnosed with diabetes between 6 and 12 months of age will have the “typical” type 1 diabetes mellitus seen in older children with positive autoantibodies against the beta cell. Autoimmune diabetes is very rare before 6 months of age and will most often be linked to specific causes.

#### IPEX Syndrome (Table 1)

Mutations of the FOXP3 gene may be responsible for enteropathy, immune dysregulation and polyendocrinopathy. It is a cause of neonatal diabetes associated with early autoimmunity directed against the beta cells of the pancreas. This diagnosis should be considered in male infants presenting diabetes associated with immune deficiency and/or severe infections. Immunosuppressant treatment can be considered (serolimus, corticosteroids) but bone marrow transplant must be considered as soon as the child's clinical condition allows. Insulin treatment will be combined with specialized nutritional management (parenteral ± enteral nutrition) before and after the transplant. It should be noted that, while correcting immune deficiencies, this will not eliminate the diabetes.

#### Down Syndrome and Neonatal Diabetes

Patients with Down syndrome (DS) resulting from trisomy 21 are more likely to have childhood diabetes mellitus. Professor Hattersley's group found 13 infants affected by DS who were diagnosed with diabetes before the age of 6 months. Trisomy 21 was seven times more likely in their PNDM cohort than in the general population (13 of 1,522 = 85 of 10,000 observed vs. 12.6 of 10,000 expected). Known PNDM genes explains 82.9% of non-DS PNDM in their work. None of the 13 DS-PNDM patients had a mutation in those genes. The conclusion from this work is that trisomy 21 is a cause of autoimmune PNDM that is not HLA associated (30).

Other mutations, such as the activating STAT3 mutations have been described which cause neonatal diabetes associated with beta-cell autoimmunity (Table 1).

## Clinical Description

There are two clinical forms of neonatal diabetes based on the duration of insulin-dependency. In the transient form, treatment may be stopped at any time from the first weeks of life to 5 years of age (4). In the permanent forms, life-long treatment is necessary.

The clinical difference between transient and permanent neonatal diabetes is not always underpinned by distinct molecular mechanisms. Abnormalities of the 6q24 locus are exclusively linked to transient neonatal diabetes. However, mutations of the *ABCC8, KCNJ11*, and *INS* genes are linked to both permanent and transient forms (17, 18, 25). Other genetic causes are associated with permanent neonatal diabetes.

Neonatal diabetes is usually diagnosed before 6 months of age. However, the age of diagnosis varies depending on genetic causes: diabetes due to a 6q24 locus abnormality appears before the age of 1 month in 93% of cases and before the age of 3 months in 100% of cases. In *ABCC8* and *KCNJ11* gene mutations, it appears before the age of 1 month in 30% of cases and between 1 and 6 months in 66% of cases (4).

At birth, patients have a birth-weight below the 10th percentile in 62% of cases (4), highlighting the crucial role of insulin secretion in fetal growth. This intrauterine growth retardation is found in all genetic groups with a greater proportion in patients with a 6q24 abnormality than those carrying a *ABCC8* or *KCNJ11* mutation (92 vs. 48%, *p* < 0.001) (4).

Half of patients with a detectable pancreas by ultrasound experience remission from the diabetes in our cohort (4). This occurs at the age of about 4 months. There is a difference depending on the genetic cause. Patients with a 6q24 locus abnormality are in remission before the age of 1 year in 97% of cases (median age 14 weeks) while remission may go as far as the age of 5 years in patients with an *ABCC8* or *KCNJ11* mutation (median age 39 weeks) (4, 31). Patients with a rare recessive mutation of the *INS* gene have remission at a median age of 12 weeks (24), whereas the majority of the *INS* gene mutations are dominant and they never go into remission. The diabetes frequently relapses (in up to 86% of cases) at the onset of puberty, probably due to the insulin resistance of puberty (4, 32). There is no difference between the genetic groups.

Depending on the genetic cause, patients with neonatal diabetes may have other clinical signs associated with diabetes (Table 1).

In neonatal diabetes with normal pancreas morphology, there are associated neurological disorders and developmental defects. Approximately 25% of patients with a mutation of the *ABCC8* or *KCNJ11* genes have neurological disorders ranging from psychomotor disorders to delayed cognitive development associated with severe epilepsy (DEND syndrome: Developmental delay, Epilepsy, and Neonatal Diabetes) (33). In addition, we have shown that when patients undergo detailed neuro-psychomotor and neuropsychological tests, an attention deficit or language disorder extending as far as dyslexia is found in 100% of cases (4).

Patients with a 6q24 locus abnormality may have developmental defects (macroglossia, umbilical hernia, cardiac malformations, renal and urinary malformations, non-autoimmune anemia, hypothyroidism with gland *in situ*) and neurological disorders (4, 11).

**Figure 2:**
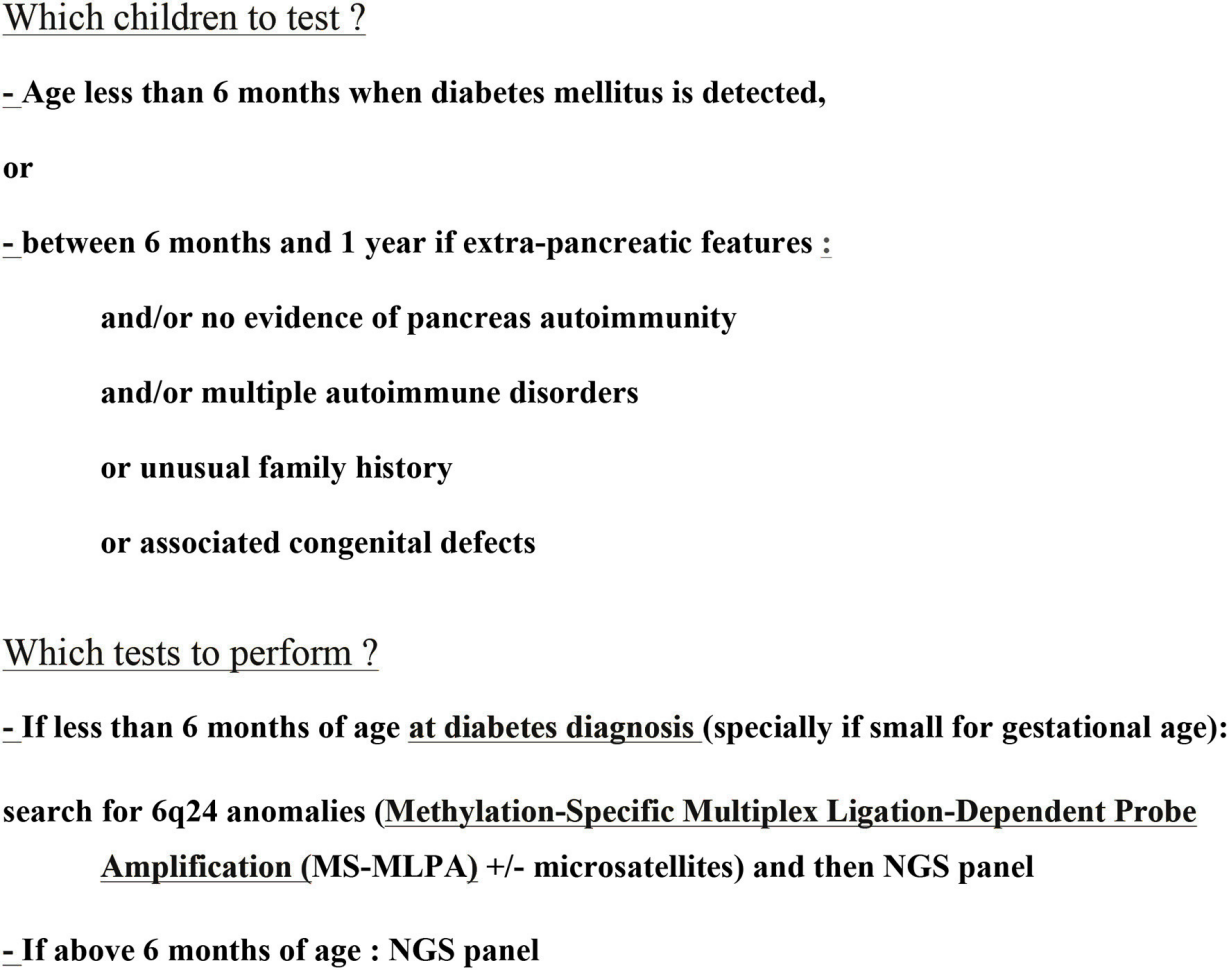
Molecular biology approach to neonatal diabetes (44).

In neonatal diabetes with abnormal pancreas morphology or with β cell destruction, the associated malformations depend on the genetic causes and are often grouped into defined syndromes (Table 1). Figure 2 illustrates a diagnostic strategy by molecular biology.

Recent long-term follow-up data in TNDM support a decrease in maximal insulin secretion capacity to both glucose and arginine stimuli that reflect low insulin mass (34). This study also showed that, regardless of the underlying genetic abnormalities or the duration of diabetes, TNDM was associated with learning difficulties at school. The high relapse rate and absence of identified predictors of relapse in TNDM suggest a need for an HbA1c assay at least every 2 years throughout childhood and for an HbA1c assay and oral glucose tolerance test every year throughout adolescence (34). During childhood, close attention should be directed to education and neurodevelopmental milestones, in TNDM patients with and without diabetes (34).

## Therapeutic Aspects

### Drug Treatment

Due to the early onset and associated delayed intrauterine retardation, patients with neonatal diabetes very often receive their initial treatment in a neonatal department. The initial treatment aims to rebalance carbohydrate metabolism. It should be started immediately following diagnosis. The treatment consists of the balance between a calorie and carbohydrate intake necessary to restore normal weight without being excessive to avoid the risk of future insulin resistance (15–18 g/kg/d carbohydrate) and sufficient insulin-based treatment to achieve the correct metabolic equilibrium. Restricting intake below the nutritional recommendations for children with low birth weight is ineffective given the physiopathology of circulating insulin deficiency.

Insulin-based treatment is difficult to manage due to the very low weight. The therapeutic margins between hypoglycemia and hyperglycemia are small, and both are harmful for neurological development of the newborn. Using an insulin pump with or without dilution of the insulin to 1:10 in 0.9% NaCl (or with a bona-fide diluent if available) can sometimes improve manageability of the insulin during the first weeks of life (35, 36). Blood glucose meters must be able to give a reliable measurement of capillary blood sugar level with the smallest possible quantity of blood (e.g., 0.3 μl blood). Few “conventional” blood glucose meters meet this criterion. Conventional capillary measurements can be done on the side edge of all the fingers, using auto-lancets offering variable pricking depths. This offers the advantage of sparing newborns' heels. An alternative is to use continuous glucose sensors, either isolated or combined with an insulin pump. In addition to enabling rapid access to interstitial blood glucose (they provide a proxy but do not actually measure the blood glucose value), they can now be coupled to the insulin pump, making it possible to activate the system to stop the insulin pump during hypoglycemia or before it occurs. They also have the advantage of minimizing the number of pricks of the skin. Used under suitable hygiene conditions, there is no increase in skin infections. It is advisable to involve experienced clinicians when treating the child and using these techniques.

Patients with *ABCC8* or *KCNJ11* mutations are treated successfully using hypoglycemic sulfonylureas, which act by binding to the regulator SUR1 subunit of the potassium channel (37) (Figure 1). The mutated channels remain sensitive to sulfonylureas in 90% of cases, having an inhibitory effect on the potassium channel of the pancreatic β cell and restoring insulin secretion in response to a meal (38). Sulfonylurea therapy appears to be safe and often successful in neonatal diabetes patients before genetic testing results are available (39). An empiric inpatient trial of sulfonylurea can be therefore considered (39). However, obtaining a genetic diagnosis remains imperative to inform long-term management and prognosis.

It has now been demonstrated that treatment with Sulfonylureas provide a better metabolic equilibrium than insulin by normalizing the HbA1c while strongly reducing the incidence of hypoglycemia in cases of neonatal diabetes with *ABCC8* or *KCNJ11* mutations. It was also shown recently that hypoglycemic sulphonylureas were able to improve neurological, neuropsychological and visuomotor impairment if they are introduced early in the child's life (33, 40, 41). Finally, a recent study has shown that it could sometimes be used successfully to replace insulin in neonatal diabetes associated with chromosome 6 methylation abnormalities (42). This emphasizes the importance of making a genetic diagnosis rapidly after diagnosing neonatal diabetes, and especially the early introduction of sulphonylureas. The clinician's aim will be to treat the child with the maximum dose that normalizes blood glucose levels (pre-prandial target: 70–120 mg/dL—post-prandial target: 100–145 mg/dL) without causing hypoglycemia, in order to optimize the drug's effect on the central nervous system. Sulphonylureas are currently only available as a 5 mg tablet and are not licensed for indications in neonatal diabetes. However, glibenclamide has recently obtained the orphan-drug indication from the European Medicine Agency (EMA) in neonatal diabetes. Unlicensed administration is currently achieved by parents through crushing and extemporaneous dilution of the tablets. However, the crushed tablets are poorly soluble in water, which may lead to variations in the dosage actually received by the child. To resolve this problem, a sulphonylurea suspension called Amglidia^R^ has demonstratable efficacy in this indication (43) and has recently obtained a European Marketing Authorization; it has been available in France under a temporary authorization for use (ATU: Autorisation Temporaire d'Utilization) since 2019. It will enable dosages to be adapted more accurately.

An Appendix added to this text describes succinctly the practical aspects of the switch from insulin injection to the glibenclamide suspension licensed in European Union for children and refers to the official summary of product characteristics for detailed information.

### Importance of the Genetic Diagnosis

Genetic analyses enables the diagnosis of monogenic diabetes in nearly 83% of diabetes diagnosed before the age of 6 months (30). This genetic diagnosis is essential as it will both influence the therapeutic treatment and make it possible to predict potential diabetes-related complications or illnesses. Genetic analyses must be carried out when diagnosing diabetes mellitus in all of the following children: age <6 months when diabetes mellitus is detected, or between 6 months and 1 year if extra-pancreatic features and/or no evidence of pancreas autoimmunity and/or multiple autoimmune disorders or unusual family history or associated congenital defects (Figure 2) (44). Testing should not be delayed until other symptoms of the disease appear or potential remission of the disease. It is also of utmost importance to identify if the sulfonylureas can be introduced successfully as high-dose sulfonylurea therapy has been shown to be an appropriate treatment for patients with KCNJ11 permanent neonatal diabetes from diagnosis. This therapy has been shown to be safe and highly effective, maintaining excellent glycemic control for at least 10 years (45).

## Conclusion

Neonatal diabetes is a model of rare human genetic disease, important in the understanding of the development and function of the pancreatic β cell, and in helping to resolve the pathophysiology of more frequent adult diabetes, such as type 2 diabetes. Neonatal diabetes is often associated with specific neuropsychological or developmental disorders of underlying genetic causes. A multidisciplinary approach is therefore essential. All clinicians called upon to treat a patient with neonatal diabetes should look for these clinical signs. Knowing the natural history and complete phenotype of this disease makes it possible, firstly, to offer patients better treatment and, secondly, to broaden the scope of genetic analyses to genes involved in the development and function of other organs. Long-term follow-up should be implemented, including for the so-called “transient” forms of neonatal diabetes.

## Author Contributions

All authors listed have made a substantial, direct and intellectual contribution to the work, and approved it for publication.

## Conflict of Interest

MP is a scientific advisor for AMMTEK. The remaining authors declare that the research was conducted in the absence of any commercial or financial relationships that could be construed as a potential conflict of interest.
