# Supplementary material for: Neonatal Diabetes Mellitus
Source: Front Pediatr. 2020 Sep 30;8:540718. doi: 10.3389/fped.2020.540718 (PMC7554616; doi:10.3389/fped.2020.540718)
Supplement: Supplementary file 1 [file Data_Sheet_1.pdf]

Appendix : Practical informations concerning the use of the glibenclamide suspension AMGLIDIA®. Source Summary of Product Characteristics.

For complete information refer to
https://www.ema.europa.eu/en/documents/product-information/amglidia-epar-product-information_en.pdf

Glibenclamide suspension therapy should be initiated by a physician experienced in the treatment of patients with very early onset diabetes.

Prescription instructions
Care should be taken when prescribing and administering AMGLIDIA to avoid dosing errors due to confusion between milligram (mg) and milliliter (mL). It should be ensured that the proper dose and strength are communicated and dispensed.

Posology

AMGLIDIA has two strengths: 0.6 mg/ml and 6 mg/ml

To avoid exceeding sodium benzoate acceptable daily dose, AMGLIDIA daily dose should not exceed 1 mL/kg/day. As a consequence, AMGLIDIA 0.6 mg/mL should not be used for posology higher than 0.6 mg/kg/day.

AMGLIDIA therapy should be initiated at 0.2 mg/kg per day in two divided doses before feeding (including bottle feeding) and increased by 0.2 mg/kg/day until insulin independence is achieved

Since AMGLIDIA is administered with an oral syringe graduated in mL, the calculated daily dose should be expressed in mL by the physician explicitly stating the strength to be used.
The syringe will be chosen (1 mL or 5mL) based on the volume in mL to be administered for each dose, as prescribed by the physician. The 5 mL syringe has to be used for volumes greater than 1 mL.
The nearest volume to the calculated one should be used.

Patients should be closely monitored by their treating physician during the titration phase.

Inpatient treatment introduction

Start AMGLIDIA at a dose of 0.2 mg/kg/day, in two administration. Give basal and bolus insulin as usual on Day 1. On Day 2, if administered sub-cutaneously, basal insulin can be removed. If on insulin pump, reduce basal rate of insulin pump by 50% and reduce further in accordance with capillary blood-glucose measurements. Throughout the transfer period, administer bolus insulin or insulin pump boluses with meals as required to maintain reasonable glycemic control. From Day 2 until the end of the titration phase, if capillary blood glucose is ≥7 mmol/L, increase AMGLIDIA by 0.2 mg/kg/day. If capillary blood glucose is < 7 mmol/L, do not increase AMGLIDIA and reduce pre-meal insulin boluses by 50%.
Pre-lunch or pre-evening meal glucose values fall more rapidly and are generally a better marker of response to AMGLIDIA.
Repeat the same protocol every day until insulin independence is achieved. As soon as insulin is discontinued, the dose of AMGLIDIA is adjusted according to capillary blood glucose.
For patients still under insulin at day 6, maintain the dose of AMGLIDIA for at least 4 weeks. This may be done as an outpatient.
Patients can be discharged when no longer requiring insulin treatment, when stable on a combination of AMGLIDIA and insulin or when stable on insulin alone.

If at the end of a 5 to 6-week period, there is no evidence of a response with insulin doses similar to those at starting, administration of doses up to 2 mg/kg/day for a week may be tried. (In rare cases, it has taken 4 months to wean off insulin completely).

If there is a clear reduction in insulin requirement at this dose of 2 mg/kg/day (reduction in insulin to at least 60% of pre-AMGLIDIA dose), then it is worth continuing a higher dose of AMGLIDIA over a prolonged period of time in selected cases.

Dosage adjustments and long-term management

As shown in the literature and in the clinical studies performed with AMGLIDIA, the average daily dose is expected to be around 0.2 to 0.5 mg/kg/day in most of the patients suffering from neonatal diabetes. Higher doses have occasionally been observed and doses up to 2.8 mg/kg/day have been successfully given without adverse reactions, according to literature. In case of a partial response on lower doses, as shown by reduced insulin requirements, a further dose increase up to 2.8 mg/kg/day may be tried in selected cases.

In children and especially in the youngest ones, a better glycemic control can be achieved when AMGLIDIA is administered 3 times or 4 times daily.

If no improvement is seen (unchanged insulin dose, similar glycaemic control and no improvement in neurology), AMGLIDIA should be discontinued.

Patients' capillary blood-glucose concentration should continue to be monitored four times a day and at bedtime, as insulin requirements may continue to fall, or AMGLIDIA may need to be titrated. Once steady state is reached, capillary blood glucose does no longer need to be daily monitored except in clinical situations at risk of metabolic unbalance (see below). In all cases, HbA1c must be monitored every three months.

Sometimes, blood-glucose concentration will fall even though the patient is on a fixed dose of AMGLIDIA. Therefore, to avoid hypoglycaemia, consideration should be given to reducing the dose of AMGLIDIA or stopping treatment.

Reduction of AMGLIDIA dose should be anticipated by the treating physician and certainly if the glucose values are going below 4 mmol/L (72 mg/dL).

It may be necessary to adjust the dosage of AMGLIDIA in patients suffering from intercurrent infections, trauma, shock or anaesthesia:

- For major surgery, insulin therapy should replace AMGLIDIA;
- Hepatic or renal dysfunction may require a reduction in dosage;
- In exceptional situations of stress (e.g. trauma, surgery, febrile infections), blood-glucose regulation may deteriorate, and a temporary change to insulin may be necessary to maintain good metabolic control.

Patients occasionally may have very high glucose values, i.e. > 20 mmol/L (> 360 mg/dL). In some cases these high glucose values seem to settle with the normal dose of AMGLIDIA. However, close monitoring of blood-glucose is required in all cases (please also refer to recommendations given under the heading "dose omission" further below) and adequate measures to restore euglycemia (e. g. application of a third daily AMGLIDIA dose or insulin) must be taken.

Dose omission

If a dose is forgotten, there is a risk of hyperglycaemia. **Blood-glucose level must be checked immediately and AMGLIDIA taken as soon as possible.** If the blood-glucose level exceeds 16.5 mmol/L, the presence of ketonuria or ketonaemia must also be checked. If ketone bodies are present, an insulin injection must be given rapidly to restore the metabolic situation. The attending specialist should then be contacted.
